# Current State and Perspectives in Population Genomics of the Common Bean

**DOI:** 10.3390/plants9030330

**Published:** 2020-03-05

**Authors:** Gaia Cortinovis, Giulia Frascarelli, Valerio Di Vittori, Roberto Papa

**Affiliations:** Dipartimento di Scienze Agrarie, Alimentari ed Ambientali (D3A), Università Politecnica delle Marche, Via Brecce Bianche, 60131 Ancona, Italy; g.cortinovis@pm.univpm.it (G.C.); g.frascarelli@pm.univpm.it (G.F.); v.divittori@staff.univpm.it (V.D.V.)

**Keywords:** population genomics, genetic diversity, evolutionary history of the common bean, adaptive selection

## Abstract

Population genomics integrates advances in sequencing technologies, bioinformatics tools, statistical methods and software into research on evolutionary and population genetics. Its application has provided novel approaches that have significantly advanced our understanding of new and long-standing questions in evolutionary processes. This has allowed the disentangling of locus-specific effects from genome-wide effects and has shed light on the genomic basis of fitness, local adaptation and phenotypes. “-Omics” tools have provided a comprehensive genome-wide view of the action of evolution. The specific features of the *Phaseolus* genus have made it a unique example for the study of crop evolution. The well-documented history of multiple domestications in *Phaseolus vulgaris* L. (common bean) and its further adaptation to different environments have provided the opportunity to investigate evolutionary issues, such as convergent evolution in the same species across different domestication events. Moreover, the availability of the *P. vulgaris* reference genome now allows adaptive variations to be easily mapped across the entire genome. Here, we provide an overview of the most significant outcomes obtained in common bean through the use of different computational tools for analysis of population genomics data.

## 1. Introduction

According to neutral theory, the great majority of evolutionary changes at the molecular level involve random fixation of selectively neutral (or nearly neutral) alleles through cumulative effects of sampling drift and under the input of novel mutations [1,2]. Neutral theory also provides the theoretical framework to be able to disentangle the roles of different evolutionary forces in the shaping of the diversity within species and populations, in order to distinguish the effects of adaptation from those of demography and population history [3,4]. The beginning of the new century can be considered the start of the population genomics era. This refers to the use of high-density markers and genome-wide sampling to identify and separate locus-specific effects (e.g., selection) from genome-wide effects (e.g., drift, gene flow and inbreeding), with the aim being to improve our understanding of population microevolution [3].

Population genomics has not just been a conceptual advance but, rather, a larger-scale approach. Its applications can address questions that have long been studied using previous tools (e.g., effective population size, population structure, phylogeography and demography) [5]. The use of novel tools and statistical tests now allows previously inaccessible issues to be addressed, such as physical mapping of adaptive variations and of molecular variants that underlie genotype fitness and relevant phenotypic variations throughout the genome [6,7,8,9].

Over the last 50 years, several techniques have been developed to assess genetic diversity and to investigate molecular evolution and phylogeny of plants. In addition to the use of classical markers (e.g., Mendelian traits), the first estimates of the levels of genetic variation within and between natural populations at multiple loci were provided in the 1970 s, using allozyme analyses [10,11]. The arrival of DNA-based markers in the late 1970 s allowed deeper investigations into genetic diversity, with the possibility to observe patterns of variation directly in DNA sequences and to quantify the number of mutations between different alleles [12]. In particular, analyses of mitochondrial DNA laid the foundation for the phylogeography field through the use of restriction fragment length polymorphism (RFLP) markers; this provided a deeper look in time at the relationships and connectivity among populations [13]. Since 1983, the applications of the polymerase chain reaction (PCR) completely revolutionized the approaches for the development and screening of genetic markers through our enhanced ability to discover mutations, which has resulted in significant advantages, such as the opportunity to examine many polymorphic loci [14].

Several PCR-based genotyping methods fall under the general category of DNA fingerprinting [15]. Among these, the discovery of simple sequence repeat (SSR) markers (also known as microsatellites), which are loci with tandem repeats of two to six nucleotide motifs, has allowed the direct scoring of both homozygous and heterozygous loci. By the end of the twentieth century, SSRs generally became the markers of choice in different population genomics studies [16]. In parallel, the development of amplified fragment length polymorphism (AFLP), which can be considered as the first class of genome-wide markers, provided the possibility to co-amplify an unprecedented number of restriction fragments without a priori information about the nucleotide sequence [17].

Direct PCR-based DNA sequencing opened the path for new approaches to genomic characterization, most notably with the discovery of the so-called third-generation markers: single nucleotide polymorphisms (SNPs). SNPs are the most abundant bi-allelic and co-dominant markers, and they are characterized by simple mutational patterns [18], with their exploitation initially made possible using Sanger sequencing technology. The advent of genotyping microarrays and, in the last few decades, the development of high-throughput sequencing methods (e.g., next-generation sequencing), further enhanced the detection and characterization of molecular markers. Next-generation sequencing platforms include such systems as: the Illumina genome analyzer, including the HiSeq, MiSeq, NextSeq and NovaSeq systems; the 454 Life Sciences FLX genome sequencer; the Thermo Fischer Scientific SOLiD, Ion Torrent and Ion Proton systems; the PacBio real-time sequencer and, more recently, the Oxford nanopore technologies. These have provided the possibility to produce millions of DNA sequence reads in a massive, parallel and high-throughput way, which has made entire genomes and transcriptomes available for population genomics studies at an exponential pace [19].

The availability of high-throughput sequencing platforms and high-density DNA markers has allowed parallel analyses of many loci on relatively large sample sizes and exploiting several statistical methods [20]. These advances have thus expanded the detection and conservation of important genetic variations to also provide a comprehensive genome-scale view of the actions of evolution [21], even in non-model organisms [22]. Moreover, the possibility to explore molecular phenotypes (i.e., metabolomics and transcriptomics data) has allowed the development of molecular evolutionary phenomics approaches [23,24].

The basic population genomics approach is characterized by four steps: sampling of individuals with different phenotypes and/or from different environments, genome-wide genotyping with high-density molecular markers, testing for outlier loci in population datasets and validation of loci that are both neutral and under selection. Neutral loci can be used to infer population demography and history, while loci putatively under selection provide adaptive information, which can be used for biodiversity conservation and evolutionary inferences [4].

With the increasing number of genetic markers available and the greater computational capacity of computers, given a sample of genes, it has also become possible to simulate the evolutionary history of a population/species under different and realistic evolutionary scenarios. Reconstruction of the genealogy that describes the descent relationships underlies the “backwards-in-time” models, for which the mathematical description is provided in the coalescent model [25]. In plant research, one of the first population genomics approaches dates back to 2005, with comprehensive studies on the whole genome of maize. In this regard, Wright et al. [26] and Yamasaki et al. [27] performed large-scale genomic screening for SNPs on 774 and 1095 randomly selected maize genes, respectively. With the aim to better understand the effects of artificial selection, these studies used a novel coalescent simulation approach and likelihood analysis, and they estimated that 2% to 4% of maize genes have been under selection during maize domestication and improvement. Moreover, the integration of quantitative trait locus (QTL) mapping and the analysis of the “selective sweep” effects allowed the target genomic regions that were under selection to be narrowed down [28].

All of these recent genomics advances described above were also widely applied in population genetics studies of the common bean, and a new epoch began for this crop. With the release of the high-quality reference genomes of the Andean G19833 [29] and the Mesoamerican BAT93 [30] genotypes, several evolutionary issues were clarified. This improved our understanding of the genome organization and its structural variations, as well as of the environmental adaptation, geographic origins, domestication and diversification of the common bean. Recent progress in population genomics of the common bean have also provided the opportunity to extend this knowledge to closely related legume species and more widely to other crops through comparative genomics studies.

*Phaseolus* spp. can be considered a unique model for the study of crop evolution. It comprises five domesticated species (*P. vulgaris*, *P. coccineus*, *P. dumosus*, *P. acutifolius* and *P. lunatus*), two of which were domesticated independently both in Mesoamerica and in the Andes (*P. vulgaris* and *P. lunatus*), which offered the opportunity to disentangle the genetic basis of the domestication process not only among species of the same genus but also between gene pools within the same species. Moreover, their recent divergence and their different mating systems make *Phaseolus* spp. an ideal system for comparative genomics studies [31]. Here we will review *P. vulgaris* studies that have addressed evolutionary questions.

## 2. Origin of the Common Bean in the Light of Different Molecular Markers

The extant genetic diversity of a population is the result of its complex evolutionary history and factors such as genetic drift, gene flow (including introgression from wild forms or closely related species), selection and mutation, along with the mating systems, are crucial in the shaping of the genetic diversity and structure of a pool of individuals.

**Figure 1:**
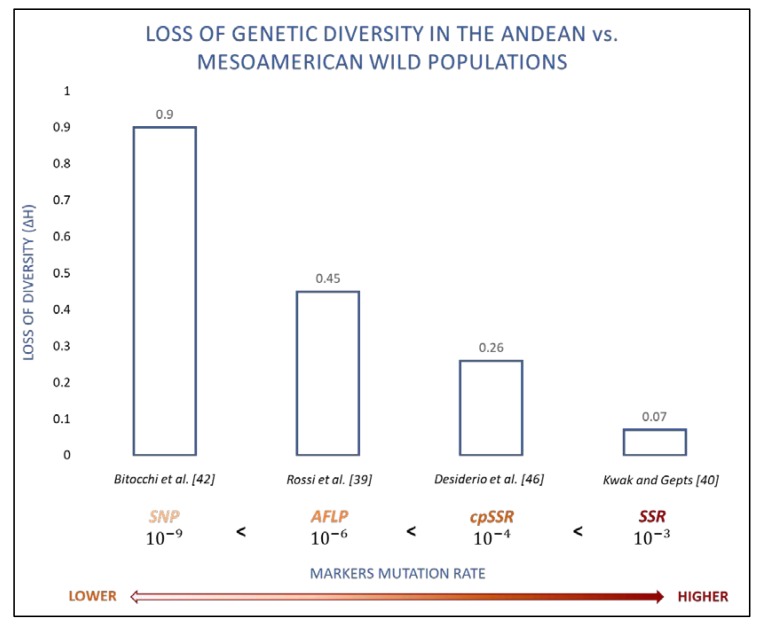
Loss of genetic diversity in the Andean versus Mesoamerican wild populations in the light of different molecular markers. SNP: single nucleotide polymorphisms, AFLP: amplified fragment length polymorphisms, cpSSR: chloroplast simple sequence repeats and SSR: simple sequence repeats.

Wild forms of *P. vulgaris* extend across the highlands of what is now Latin America, between Northern Mexico and Northwestern Argentina [32]. These are characterized by three main eco-geographic gene pools: Mesoamerican and Andean, which are the major gene pools, with both including wild and domesticated forms, and the population from Northern Peru-Ecuador (PhI), which has a relatively narrow distribution of wild individuals [33]. Phaseolin data [34,35], allozymes [36] and multi-locus markers [37,38,39,40] have together confirmed the structure of the diversity of the gene pools of the common bean, and have often highlighted the higher genetic variability of the Mesoamerican gene pool compared to the Andean one. Indeed, Rossi et al. [40] used a large set of AFLP markers to dissect out the internal structure within both the Mesoamerican and the Andean gene pools, and they always detected a higher proportion of polymorphic loci in the wild forms compared to the domesticated ones, with the Mesoamerican gene pool being much more diverse and structured compared to the Andean population. Rossi et al. [40] compared their AFLP data with SSR data from Kwak and Gepts [41], and they noted that the differences in genetic diversity between the Mesoamerican and Andean wild gene pools were highly associated with the mutation rates of the molecular markers; the higher the marker mutation rate, the lower the differences between the Mesoamerican and Andean gene pools (Figure 1). Based on this, and assuming the bottleneck model proposed by Nei et al. [42], Rossi et al. [40] suggested that only the Mesoamerican origin of *P. vulgaris* could explain the contrasting patterns of diversity for different molecular markers when comparing the Andean and Mesoamerican gene pools. This hypothesis was supported and strongly consolidated by Bitocchi et al. [43], who showed a loss of nucleotide diversity (Lπ) of 90% in the Andean wild population compared to the Mesoamerican population (Figure 1).

Markers that differ in their mutation rates can highlight very different patterns of molecular diversity in the same species or population; this is because the number of generations needed for mutations to allow the recovery of the genetic diversity after a bottleneck is expected to be close to the reciprocal of the mutation rate of the markers [42,44,45]. Thus, the lower mutation rates of SNPs (for the *Fabaceae* family, this was estimated to be ∼6.1 × 10^−9^ mutations per base pair per generation [46]) compared to other types of markers allowed Bitocchi et al. [43] to detect the occurrence of the Andean bottleneck with much greater resolution. Indeed, they detected a loss of genetic diversity of about two-fold, three-fold and 13-fold those observed in a comparable sample of *P. vulgaris* genotypes using AFLP (45%) [40], chloroplast (cp) SSRs (26%) [47] and SSRs (7%) [41], respectively (Figure 1).

**Figure 2:**
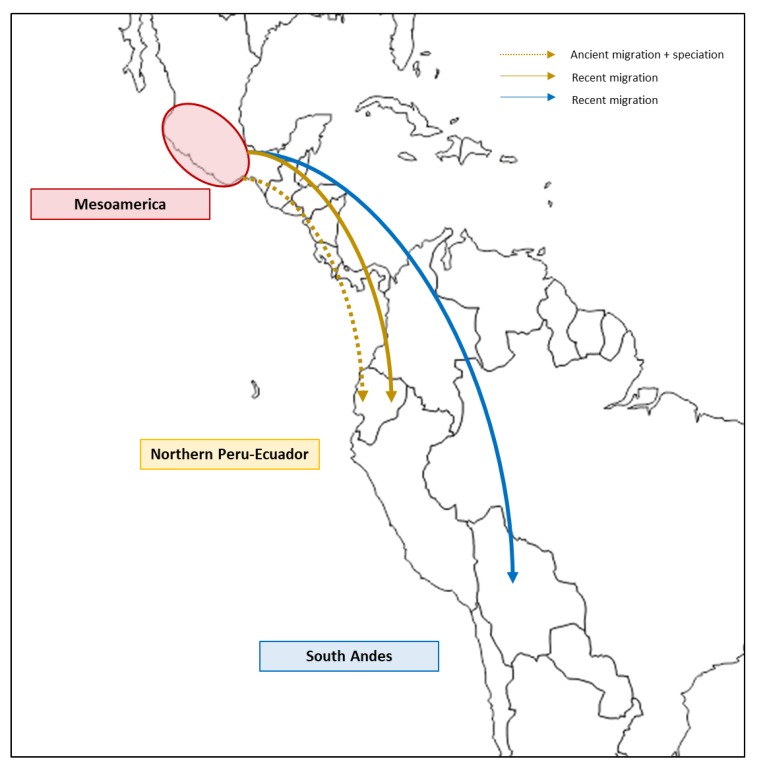
Graphical representation of the two different evolutionary hypotheses for wild *P. vulgaris* migration in America.

The Mesoamerican origin hypothesis was also supported by additional data from Bitocchi et al. [43], who were the first to report a clear-cut population structure into four different groups for the wild Mesoamerican accessions. Moreover, their phylogenetic analysis revealed that both the Andean and the Northern Peru-Ecuador wild accessions were strongly related to two distinct Mesoamerican groups that were located in a wide area of Central Mexico. Thus, both the Andean and Northern Peru-Ecuador gene pools appeared to have originated through different migration events from the Mesoamerican populations of Central Mexico (Figure 2), as also confirmed by the work of Schmutz et al. [29] and supported by the approximate Bayesian computation analysis performed by Ariani et al. [48].

Recently, Rendón-Anaya et al. [49] proposed the hypothesis of a slightly different evolutionary history, with the suggestion that the introduction in Northern Peru-Ecuador originated from an ancestral form and occurred much earlier than the diversification of *P. vulgaris* within the *Vulgaris* group (Figure 2). They analyzed both the chloroplast and nuclear genomes by sequencing 18 *P. vulgaris* accessions that spanned the three gene pools (eight wild, two domesticated Mesoamerican accessions; one wild, two domesticated Andean accessions and five Northern Peru-Ecuador accessions) to reconstruct the phylogenetic relationships of this species. While they supported the Mesoamerican origin of common bean, Rendón-Anaya et al. [49] also proposed an early dissemination and speciation event in Mesoamerica before the split into the two current major gene pools (i.e., Mesoamerican and Andean). Using a maximum-likelihood approach, they performed a phylogenetic analysis based on genome-wide SNPs and on a 55-kb chloroplast genome fragment. They observed two distinct clades, one that included all of the wild Peru-Ecuador accessions and another that included all of the other *P. vulgaris* genotypes. These results led them to conclude that the Northern Peru-Ecuador population evolved from a speciation event that occurred before the separation of the Andean gene pool from the Mesoamerican gene pool. However, considering the results obtained by Desiderio et al. [46], more genotypes (preferably as wild accessions) should be analyzed to validate this hypothesis.

## 3. Domestication of the Common Bean

Phenotypic and genotypic information based on a variety of methods coherently support the occurrence of two independent domestication events, one in Mesoamerica and the other in the Andes, where the two major domesticated gene pools originated [35,40,41,50,51]. The genetic routes towards domestication were also confirmed recently by Schmutz et al. [29], who performed pooled resequencing of 160 wild and domesticated accessions from the centers of origin, as well as releasing the first high-quality reference genome of *P. vulgaris*.

### 3.1. Mapping Domestication Traits

Domestication resulted in several phenotypic and genetic changes between the domesticated forms and their wild ancestors (i.e., the domestication syndrome), such as differences in growth habit and photoperiod sensitivity; variations in shape, color and size of the edible parts and reduction or loss of seed dormancy and seed shattering. Starting from the pioneering work of Koinange et al. [52] to date, molecular variations and QTLs associated with the modulation of domestication traits have been mapped and partially characterized [53], with recent major advances towards the identification of the genes responsible for seed shattering [54,55,56], determinate/indeterminate growth habits [57] and seed color [58].

One of the most important targets of domestication in the common bean was the seed shattering trait. Wild common bean is characterized by high seed shattering, which is crucial for propagation of the progeny and to ensure high fitness of the genotypes. Conversely, the domesticated forms have partially or completely lost this seed dispersal, as lower dehiscence ensures reduction of yield losses in the field [59,60]. Rau et al. [54] used an introgression line-mapping population and proposed a model with a major locus associated to the complete loss of pod shattering, which was localized to the distal part of chromosome Pv05, plus additional QTLs hypostatic to the major QTL, at which the cumulation of wild alleles increases the level and mode of seed shattering (i.e., number of shattered and twisted pods per plant). The major QTL was recently confirmed by Parker et al. [55], who performed association mapping on a panel of 208 Andean accessions. In the model proposed by Rau et al. [54] for modulation of seed shattering, at least other two loci on chromosomes Pv05 and Pv04 were proposed, and overall, the model explained 72.4% of the phenotypic variance for this trait. Additional loci with minor effects on chromosomes Pv09, Pv04 and Pv06 were proposed to explain the variability associated with the level of seed shattering [54]. The multi-locus control of seed shattering was confirmed by Parker et al. [55], who mapped loci for seed shattering also on chromosomes Pv03, Pv08 and Pv09. Several genes have been proposed to be responsible for the genetic control of the pod-shattering trait, but additional analyses are needed to further narrow down the list of candidates. With regards to the growth habit, Repinsky et al. [57] identified the functional orthologue to *AtTFL1* (*Terminal Flower 1*) in the common bean (*PvTFL1y*). The co-segregation of *PvTFL1y* with the *fin* locus [52] for the determinate growth habit, the strong decrease in mRNA abundance associated with two haplotypes at *PvTFL1y* locus and the rescue of the indeterminate phenotype in the *tfl1* mutant in *Arabidopsis* with the wild allele of *PvTFL1y* allowed Repinsky et al. [57] to establish that *PvTFL1y* gene controls the determinate/indeterminate habitus. McClean et al. [58] recently characterized the molecular structure of the gene responsible at the *P* locus for the presence/absence of seed color in the common bean, and they validated its function through virus-induced gene silencing. They identified four alleles at the basis of the pigmented seeds phenotype, while several independently derived *p* alleles for white seeds were detected, which suggested that a convergent evolution mechanism is at the basis of the white-seed phenotype. Recent and future advances in the development of population genomics tools and statistical approaches will have a crucial role in shedding light on the genetic basis of pod shattering and on other domestication/diversification traits.

### 3.2. Signature of Selection

As previously described, population genomics aims to disentangle the effects of selection from those of other evolutionary forces through analysis of the aberrant patterns of DNA polymorphisms and assuming a neutral scenario. The domestication process is usually associated with a reduction in genetic diversity [40,50,61,62] and with an increase in divergence between wild and domesticated populations, due to demographic factors that affect the entire genome and to natural and artificial selection at target loci [63]. Moreover, considering the parallel domestication in the Andes and Mesoamerica, Bitocchi et al. [50] estimated a three-fold greater reduction in genetic diversity between the wild and domesticated Mesoamerican pools, with respect to the equivalent comparison in the Andean pool. These data can be explained as a consequence of the bottleneck that occurred in the wild Andean germplasm, which impoverished the genetic diversity before domestication and resulted in a lesser effect of the subsequent domestication in the Andes.

Papa et al. [64] used AFLP markers to identify several associations between the map locations of various domestication genes and QTLs and the regions of high divergence between the wild and domesticated genotypes. The potential of the use of population genomics in the common bean was also demonstrated by Papa et al. [65], where they used pooled DNA samples and analyzed 2506 AFLP loci to identify a large portion of the genome (16%) that had been affected by the domestication process, with many markers under selection associated to known loci for the domestication syndrome traits.

Bellucci et al. [23] exploited the potential of RNA sequencing (RNA-seq), which combines information from nucleotide diversity and gene expression, and demonstrated for the first time that common bean domestication in Mesoamerica was characterized not only by a significant reduction in the nucleotide diversity but also by deep impact on the architecture of gene expression and co-expression at the whole transcriptome level. In more detail, Bellucci et al. [23] adopted an approach based on de novo assembly of a reference transcriptome, and they mapped on it the RNA-seq data from 21 inbred genotypes: 10 wild and eight domesticated Mesoamerican genotypes, with one wild and two domesticated Andean genotypes as controls. The final dataset of 188,107 SNPs distributed across 27,243 contigs was used to study the domestication process of the common bean in Mesoamerica. Bellucci et al. [23] identified signatures of selection on contigs from RNA-seq data by testing the significance of two ad-hoc statistical indices and using a coalescent simulation approach that considered the absence of selection during domestication. Taking into consideration the demographic parameters available from previous studies [61,66], Bellucci et al. [23] revealed that 9% of the contigs were actively selected during common bean domestication and that the selection in these contigs induced further reductions (26%) in the diversity of gene expression. Generally, genes that are putatively under selection show greater genetic diversity in the wild alleles compared to the domesticated alleles. Indeed, most of the contigs affected by selection in Bellucci et al. [23] were monomorphic in the domesticated gene pool and polymorphic in the wild germplasm. However, diversifying selection was also detected, which was reflected in a small fraction (2.8%) of the contigs in which the wild forms were fixed monomorphic, while the domesticated accessions were highly polymorphic. In addition, looking at differentially expressed contigs, down-regulation was observed mainly in the domesticated accessions, compared to the wild accessions, which indicated the occurrence of loss-of-function mutations. These results suggested that domestication increased the functional diversity at a few target loci in parallel with an overall reduction in genetic diversity at the transcriptome-wide level. The results of Bellucci et al. [23] can be imputed to novel mutations that were selected for expansion and adaptation to new environments and agro-ecological growth conditions.

In parallel with the release of the common bean reference genome, Schmutz et al. [29] performed the first genome-wide analysis that considered both of the gene pools. They dissected out the effects of domestication at the genome-wide level by comparing wild and landrace accessions across 10-kb/2-kb sliding windows in the top 90% of the empirical distribution of the population for both π_wild_/π_landrace_ ratios and F_ST_ values. They analyzed the F_ST_ distribution and the loss of nucleotide diversity, and they defined genes and genomic regions under selection during domestication in each of the gene pools. Interestingly, only 7.2 Mb of the genome putatively under selection were shared between the Mesoamerican and Andean groups. Moreover, out of 1835 Mesoamerica and 748 Andean candidate genes, only 59 were common between the two domestication events.

**Figure 3:**
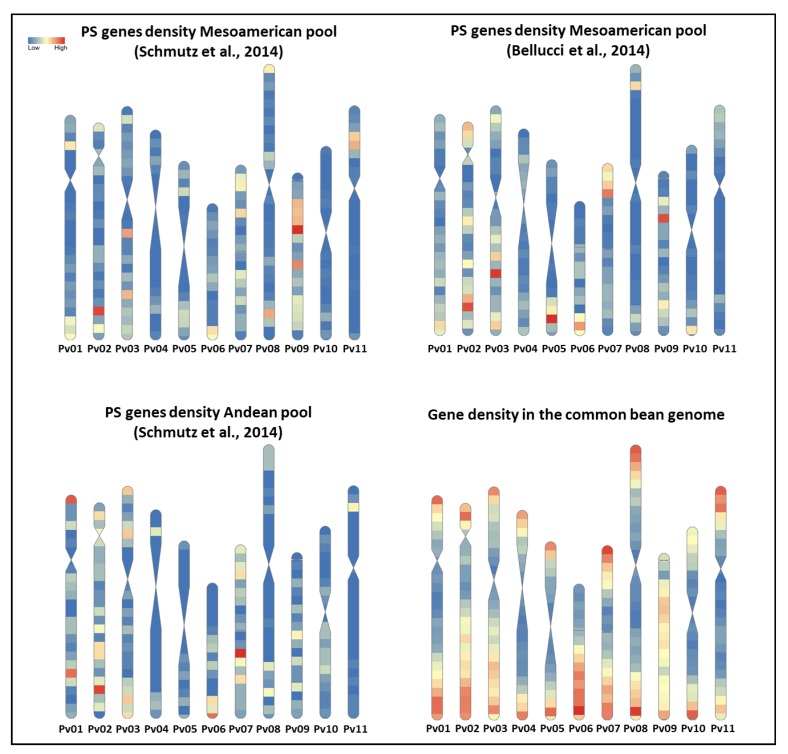
Genome-wide distribution of putatively under selection (PS) genes. **Top left**: distribution of PS genes in the Mesoamerica gene pool, according to Schmutz et al. [29]. **Top right**: distribution of PS genes in the Mesoamerica gene pool, according to Bellucci et al. [23]. **Bottom left**: distribution of PS genes in the Andean gene pool, according to Schmutz et al. [29]. **Bottom right**: gene density across the entire genome. Gene density is highlighted according to the color intensity in the legend at a 2-Mb window scale.

Out of the total of 2364 PS contigs identified by Bellucci et al. [23], Di Vittori [56] identified 1642 PS genes that are the reference for 1935 PS contigs. According to the new mapping for the PS contigs of Bellucci et al. [23] and to the available information from Schmutz et al. [29], Figure 3 shows the genome-wide PS gene density in the Mesoamerican [23,29] and Andean [29] gene pools. These maps were constructed using the RIdeogram package [67] in R. Interestingly, for all of the dataset, we identified only a few common regions with higher densities of PS genes as, for example, at the end of chromosomes Pv02 (~42–44 Mb) and Pv06 (from ~26 Mb to the end of the chromosome) and at the beginning of chromosomes Pv07 and Pv08. Moreover, in both of these studies, genomic regions with high-density of PS genes specific for the Mesoamerica gene pool were identified as, for example, at the end of chromosome Pv01 and around 10-12 Mb on Pv09. With regard to these regions, Parker et al. [55] recently identified a QTL for seed shattering at the beginning of chromosome Pv08, while the orthologue to *ATIND* [68] (*PvIND* in [69]) was localized to 44 Mb on Pv02 in the same region where the *St* locus for the pod string presence was mapped [52]. Interestingly, the major growth habit gene in the common bean, Phvul.001G189200 (*PvTFL1y*) [57], is located at ~45 Mb on Pv01, close to regions with relative high densities of selection signatures in the Mesoamerican gene pool. In addition to these regions, Figure 3 also highlights differences between Mesoamerican and Andean pools for the PS gene location, according to the data of Schmutz et al. [29], which agrees with the occurrence of at least two parallel domestication events.

However, a similar density distribution of PS genes can be observed between the Mesoamerican [23] and Andean [29] gene pools with respect to the analysis of Schmutz et al. [29] of the Mesoamerican gene pool, as for example for the chromosome Pv02 (Figure 3). This last observation might suggest that integration of different datasets and approaches (e.g., genomic, transcriptomic and metabolomic analyses) can allow comparative studies and the identification of uncovered selection signatures, although, at the same time, discordance between different analyses might arise from the different statistical approaches and sampling issues adopted for the detection of the selection signatures. Thus, deeper investigations are needed to understand the convergent evolution of domestication traits in common bean.

## 4. Diversification and Adaptation of P. Vulgaris to Different Agro-Ecological Conditions

As mentioned above, the wild common bean originated in Mesoamerica and, through migration events, it subsequently expanded from Northern Mexico to Northwestern Argentina, to encompass some 70 latitudinal degrees and elevations between 500 and 2000 m a.s.l [70,71,72]. This broad geographic distribution indicated that, through adaptive evolution, *P. vulgaris* colonized different agro-ecological locations compared to its area of origin. Several population genomics approaches can uncover the basis of genetic adaptation to different environments by looking at specific signatures at the genomic sequence level (e.g., outlier-loci detection strategy) or by looking for associations between genomic loci, phenotypic traits and environmental data [73,74].

Landscape genomics is an emerging discipline that combines population genetics, landscape ecology and spatial analytical techniques to identify environmental factors that have shaped adaptive variations that underlie local adaptation [4,75]. The number of landscape genomics studies has risen exponentially since 2003 [76], and among these, a study conducted by Rodriguez et al. [77] was the first particularly thought-provoking example of the potential of this method in *P. vulgaris*. In more detail, Rodriguez et al. [77] analyzed correlations between molecular markers and ecological variables at a continental scale. They used 131 SNPs in a population of 577 accessions (417 wild and 160 domesticated) that encompassed the wide geographic distribution of the common bean in America, with the aim being to examine the genetic–spatial patterns of the wild common bean. They reported the existence of well-defined wild genetic groups and variable degrees of diversity in both the Mesoamerican and Andean gene pools. Therefore, they investigated the spatial distribution of diversity using Mantel tests and multivariate analysis (using genetic and geographic information), which allowed them to determine the correlation between genetic and geographic distances. These analyses highlighted the presence of global structures (i.e., geographically closer individuals were also more genetically similar), which suggested that the effects of migration and genetic drift overlapped with selection effects in the same direction, with the consequent divergent selection as a result of local adaptation. Geographic and environmental data were combined with genetic diversity data to separate the effects of geography from those of ecology, and they reported a total of 26 loci (19.8%) that were putatively under selection for adaptation. Among these, different loci were shown to have compatible functions with adaptation features, such as chilling susceptibility, cold acclimation and mechanisms related to drought stress.

Recently, Ariani and Gepts [78] performed a similar analysis on 246 wild common bean accessions using a larger number of markers (~20,000 SNPs) that were widely distributed across the genome. Ariani and Gepts [78] coupled 19 bio-climatic variables with genome scan analysis for selection and genome-wide association analysis to identify which gene pools/genes were putatively under adaptive selection by temperature. Among the candidate genes identified by Ariani and Gepts [78], *Phvul.002G143100* appears to be particularly interesting; indeed, in the *Arabidopsis thaliana* model system, the homologous gene (*AtGRDP2*) is involved in flowering-time regulation, and its overexpression results in significant reduction in days to flowering [79]. The timing of important phenological stages is one of the most crucial diversification traits, in as much as it reflects the adaptation of a species through the tailoring of vegetative and reproductive growth phases to local climatic effects.

*P. vulgaris* became widespread not only in the Americas, as its cultivation extended worldwide, and it became the most important grain legume for direct human consumption [80]. The dissemination and introduction of the common bean into the Old World, as well as for other New World crops such as tomato, maize, squash, potato and tobacco, occurred after the 1492 voyage of Christopher Columbus. To investigate the evolutionary patterns of the common bean far from the New World, Angioi et al. [81] analyzed 94 and 307 *P. vulgaris* accessions from the Americas and Europe, respectively. Several studies based on molecular and biochemical markers [69,81,82] reported that the European common bean populations include both Mesoamerican and Andean forms, and that the Andean germplasm was the most represented in Europe, even though the proportions of these gene pools can significantly differ across countries. Angioi et al. [81] also estimated that 44.2% of the European landraces derived from at least one hybridization event between Mesoamerican and Andean forms, which demonstrated the fundamental role of hybridization and recombination in the origin of the European common bean gene pool. This hybridization was promoted by the breakdown of the spatial isolation between the Mesoamerican and Andean accessions after their introduction into Europe and had a crucial impact on the maintenance of genetic diversity and common bean adaptation to highly variable environments. Novel combinations of genes/genomic regions probably arose in Europe after the introduction of the common bean and during its dissemination, on which adaptive selection acted (i.e., adaptive introgression). The new “-omics” technologies can help to fine-tune the molecular basis of these adaptation strategies, an aspect that is ongoing in the BEAN_ADAPT Project (funded through the second ERA-CAPS call; ERA-NET for Coordinating Action in Plant Sciences). This project is based on a multidisciplinary approach (i.e., genomics, transcriptomics, metabolomics, plant physiology, population/quantitative genetics and biochemistry) with the aim to extend the genetic basis of the phenotypic adaptation of *P. vulgaris* and its sister species *P. coccineus* in Europe and outside of their centers of origin.

## 5. Conclusions

Population genomics research is providing a more complete picture of genetic parameters across the entire genome in both model and non-model species [83]. The plummeting costs of DNA sequencing make genotyping feasible for hundreds to millions of individuals and loci and also allow the study of variations in gene expression, epigenetics and proteins. Furthermore, the combination of genome-wide data from sequencing tools, with improved coverage and resolution of metabolomic platforms, also allows mapping of several metabolites (mQTL mapping) [84]. For instance, Beleggia et al. [24] investigated for the first time the effects of selection on the accumulation of 51 primary metabolites and their relationships in the kernels of three *Triticum turgidum* L. subspecies, and they revealed domestication-associated changes in metabolite contents and in the metabolic correlation networks. Few metabolomics studies have been conducted so far in population genomics of the common bean [85]; however, even though there are sometimes still limits in the immediate translation of genetic variation into metabolic diversity [86], the integration of metabolic profiling with other “-omics” data might be highly effective for functional gene identification and elucidation of the common bean demographic history. One interesting study was carried out recently by Perez de Souza et al. [85], who combined a new approach for the annotation of specialized metabolites with transcriptomic sequencing data and phylogenetic analyses for several genotypes of *P. vulgaris* that belong to the Mesoamerican and Andean gene pools. Their data show that three classes of metabolites (i.e., hydroxycinnamates, flavonoids and triterpene saponins) accumulated differently across their accessions; moreover, the creation of a multi-omics dataset allowed them to identify with precision and accuracy a set of candidate genes that were responsible for important agronomical and ecological traits.

The main potentiality of population genomics approaches in *P. vulgaris* is emerging with the unraveling of the genetic bases of common bean domestication and adaptation to different environmental conditions. The discovery of advantageous genetic variants is fundamental not only to clarify the evolutionary history of a certain population but also to determine the heritability of simple and complex traits in order to design successful breeding strategies. Indeed, identification of the genetic architecture of plant adaptation to different environmental conditions appears to be a major element to address crucial societal challenges, such as mitigation and adaptation to climate changes [87]. Moreover, an excellent example of the potential population genomics approach was offered by Exposito-Alonso et al. [88] for the *A. thaliana* model system. As well as improving our knowledge of the genomic basis of past selection and adaptation to specific agro-ecosystems, Exposito-Alonso et al. [88] were able to build genome-wide environmental selection models to predict how evolutionary pressures on species will work in inaccessible environments or even under future hypothetical climates [9].

Figure 1 shows the critical role of marker mutations in describing the diversity of plant populations; the higher the mutation rate, the lower the loss of diversity detectable. The occurrence of a bottleneck in the Andes before domestication was recovered from more quickly by markers with a high mutation rate compared with markers showing lower rates of mutation. For this reason, the lower mutation rate characteristic of SNP markers allowed Bitocchi et al. [43] to detect the effects of the bottleneck on the genetic diversity of the Andean wild germplasm with much higher resolution (Lπ = 90% compared to the Mesoamerican wild gene pool), which confirmed the Mesoamerican origin of the common bean.

Figure 2 shows the Mesoamerican origin of wild *P. vulgaris* proposed by Bitocchi et al. [43] consists of the hypothesis that the Andean and Northern Peru-Ecuador wild common bean populations originated from two independent migrations of the Mesoamerican wild population (Figure 2, blue and yellow solid arrows), which occurred about 110,000–165,000 years ago, prior to the domestication of the species. This hypothesis has also been supported by subsequent studies [29,47,66], and recently, it was confirmed by approximate Bayesian computation analysis [48]. Subsequently, two parallel and independent domestication events in Mesoamerica and in the Andes gave rise to the formation of the current two major domesticated gene pools. Rendón-Anaya et al. [49] proposed the hypothesis of a slightly different evolutionary history, supporting the occurrence of two migration events at different times. In particular, compared to the previous hypothesis, they suggested that the introduction of the wild ancestor “*Phaseolus protovulgaris*” into Northern Peru-Ecuador from Mesoamerica occurred much earlier (ancient migration, 0.9 Mya for plastid markers; Figure 2, dashed yellow arrow) than the diversification of *P. vulgaris* within the *Vulgaris* group.
